# Metabolic Reprogramming Mediates Delayed Apoptosis of Human Neutrophils Infected With *Francisella tularensis*

**DOI:** 10.3389/fimmu.2022.836754

**Published:** 2022-05-25

**Authors:** Samantha J. Krysa, Lee-Ann H. Allen

**Affiliations:** ^1^ Inflammation Program, University of Iowa, Iowa City, IA, United States; ^2^ Molecular Medicine Program, University of Iowa, Iowa City, IA, United States; ^3^ Iowa City VA Health Care System, Iowa City, IA, United States; ^4^ Department of Medicine, Division of Infectious Diseases, University of Iowa, Iowa City, IA, United States; ^5^ Department of Microbiology and Immunology, University of Iowa, Iowa City, IA, United States; ^6^ Harry S. Truman Memorial VA Hospital, Columbia, MO, United States; ^7^ Department of Molecular Microbiology and Immunology, University of Missouri School of Medicine, Columbia, MO, United States

**Keywords:** glycolysis, glycogen, neutrophils (PMNs), apoptosis, immunometabolism, *Francisella tularensis*

## Abstract

Neutrophils (polymorphonuclear leukocytes, PMNs) have a distinctively short lifespan, and tight regulation of cell survival and death is imperative for their normal function. We demonstrated previously that *Francisella tularensis* extends human neutrophil lifespan, which elicits an impaired immune response characterized by neutrophil dysfunction. Herein, we extended these studies, including our transcriptional profiling data, and employed Seahorse extracellular flux analysis, gas chromatography-mass spectrometry metabolite analysis, flow cytometry and several other biochemical approaches to demonstrate that the delayed apoptosis observed in *F. tularensis*-infected neutrophils is mediated, in part, by metabolic reprogramming. Specifically, we show that *F. tularensis*-infected neutrophils exhibited a unique metabolic signature characterized by increased glycolysis, glycolytic flux and glucose uptake, downregulation of the pentose phosphate pathway, and complex glycogen dynamics. Glucose uptake and glycolysis were essential for cell longevity, although glucose-6-phosphate translocation into the endoplasmic reticulum was not, and we identify depletion of glycogen as a potential trigger of apoptosis onset. In keeping with this, we also demonstrate that ablation of apoptosis with the pan-caspase inhibitor Q-VD-OPh was sufficient to profoundly increase glycolysis and glycogen stores in the absence of infection. Taken together, our data significantly advance understanding of neutrophil immunometabolism and its capacity to regulate cell lifespan.

## Introduction

Polymorphonuclear leukocytes (PMNs, neutrophils) comprise the majority of circulating white blood cells within the human body and are produced at a rate of approximately 100 billion per day (1). As one of the first immune cells to be recruited to infection sites, neutrophils are a vital component of the immune system that rapidly identify, engulf, and eradicate invading microbes (1–3). Neutrophils have a uniquely short lifespan of 18-24 hours in circulation before undergoing constitutive apoptosis, and disruption of this tightly regulated, pre-programmed cell death mechanism disrupts neutrophil function and capacity to resolve infection (4, 5). Mature neutrophils are also metabolically distinctive, as they rely primarily on glycolysis for energy production (6). Although it is known that the idiosyncratic lifespan and metabolism of neutrophils each contribute distinctly to supporting neutrophil function, the extent to which neutrophil metabolism influences lifespan, or vice versa is not well understood (4, 7).

Constitutive apoptosis is tightly regulated and requires global changes in PMN gene expression (4, 8). Overall, cell viability and death are governed by the relative abundance of pro-survival and pro-apoptosis BCL-2 family proteins as well as Inhibitor of Apoptosis Proteins (IAPs) and calpastatin (9). The key event in early apoptosis is permeabilization of the outer mitochondrial membrane by pro-apoptosis BCL-2 proteins BAX and BAK. Thereafter, cytochrome *c* released into the cytosol initiates apoptosome formation for activation of caspase-9, which in turn activates caspase-3 for execution of cell death. In healthy cells mitochondrial integrity is maintained by pro-survival BCL-2 proteins MCL-1 and BCL2A1 which block translocation of BAX and BAK. At the same time, IAPs such as XIAP prevent processing activation of procaspase-9 and procaspase-3 by direct binding. Additional layers of regulation are provided by cIAP-1 cIAP-2, calpastatin, cyclin-dependent kinases, extrinsic pathway regulators and signaling mediated by growth factor receptors and inflammatory mediators (4, 9). As survival factors are short-lived, continued expression of the encoding genes is critical for PMN viability, but the internal signal that tips the balance toward apoptosis in aging PMNs is unknown (4, 9).

*Francisella tularensis* is a Gram-negative facultative intracellular coccobacillus, the causative agent of the zoonotic disease tularemia, and one of the most infectious pathogens known (10–15). One of few bacteria capable of parasitizing neutrophils, *F. tularensis* evades elimination *via* a multifaceted strategy that includes inhibition of NADPH oxidase assembly and activity, followed by phagosome escape and replication in the cytosol (16, 17). At the same time, *F. tularensis* inhibits neutrophil apoptosis and accumulation of dysfunctional neutrophils at the infection site contributes to disease exacerbation rather than resolution (18–21). These properties are shared by *F. tularensis* subspecies *tularensis* (type A) strains that are exclusive to North America as well as *F. tularensis* subspecies *holarctica* (type B) strains that are found throughout the Northern Hemisphere (16). Although it is unequivocal that *F. tularensis* extends neutrophil lifespan by delaying apoptosis, the mechanisms enabling infected cells to override these highly conserved, tightly regulated apoptosis programs are still incompletely defined (22–24).

Metabolic regulation of inflammation and immune cell function is a rapidly growing field of study. Although most studies to date have focused on macrophages and T lymphocytes, distinct metabolic states of neutrophils are beginning to be described (7, 25–30). Thus, recent data demonstrate that neutrophil metabolism can be reprogrammed and that these adaptations contribute directly to elimination of infection or disease progression in a context-specific manner (25–27, 31, 32). On the other hand, there are limited data regarding potential links between changes in neutrophil behavior elicited by metabolic reprogramming and cell lifespan. In previous work, our laboratory demonstrated that *F. tularensis* significantly extends human neutrophil lifespan *via* effects on apoptosis pathway signaling and changes in gene expression leading to upregulation of prosurvival factors such as XIAP, calpastatin and BCL2A1 that inhibit caspase activation and sustain mitochondrial integrity (23, 24). We undertook the current study as our transcriptional profiling data suggested that *F. tularensis* may also manipulate neutrophil metabolism. Herein, we demonstrate that *F. tularensis* elicits a distinct metabolic program that is defined by dynamic changes in glycolysis and glycogen abundance that are essential for cell longevity. In addition, we also show that pan-caspase inhibition can alter metabolism in the absence of infection. Collectively, these data advance understanding of PMN metabolic plasticity and support the hypothesis that metabolism and PMN lifespan are intimately linked.

## Materials and Methods

### Cultivation of Bacteria

*F. tularensis* subspecies *holarctica* live vaccine strain (LVS) was inoculated onto Difco cysteine heart agar (BD Biosciences, East Rutherford, NJ) supplemented with 9% defibrinated sheep blood (Hemostat Labs, Dixon, CA) and grown at 37°C in 5% CO_2_ for 48-72 hr. Bacteria were transferred from the plate into 1 ml of sterile Hank’s Balanced Salt Solution (HBSS) containing divalent cations (Thermo Fisher Scientific, Waltham, MA) and quantified by measurement of absorbance at 600 nm. Broth cultures were started at an OD_600_ of 0.01 in 5 ml pH 6.8 Bacto brain heart infusion (BHI) broth (BD Biosciences) in a 50 ml conical tube. All broth cultures were incubated at 37°C in 5% CO_2_, shaking at 200 RPM and grown to mid-exponential phase either by 1) being incubated for 12 hr, followed by immediate harvest; or 2) by being incubated for 15-17 hr, diluted to an OD_600_ of 0.200 in 5 ml BHI broth and incubated for 2–4 more hr prior to harvest. Mid-exponential growth phase bacteria were pelleted at 12,000 RPM for 2 min, washed once in 1 ml HBSS with divalent cations and quantified by measurement of absorbance at 600 nm.

### Ethics Statement

Heparinized, venous blood was obtained from healthy adult volunteers who provided written informed consent according to protocols approved by the Institutional Review Board for Human Subjects at the University of Iowa (#201609850 and #200307026).

### Isolation of Neutrophils From Human Blood

Neutrophils were isolated *via* sequential dextran sedimentation (Pharmacosmos, Holbæk, Denmark), density separation through a Ficoll-Hypaque gradient (GE Healthcare, Chicago, IL) and hypotonic lysis of erythrocytes (33). This method routinely yielded >95% neutrophil purity.

### Neutrophil Ultrapurification

As indicated, certain experiments utilized ultrapure PMNs. To achieve this, neutrophils, isolated as described above, were counted and centrifuged at 1,100 x g for 5 min. Cells were resuspended to 5 x 10^7^/ml in PBS without cations supplemented with 2% fetal bovine serum (Hyclone Laboratories, Pittsburgh, PA) and 1mM EDTA and transferred to 5 ml round-bottomed polypropylene tubes in 0.25-2.5 ml aliquots. Neutrophils were purified using the EasySep™ Human Neutrophil Isolation Kit from Stem Cell Technologies (Vancouver, Canada), according to manufacturer’s instructions. Following the ultrapurification process, neutrophil purity was assessed by staining cells (1 x 10^6^ cells/staining condition) with anti-CD15-Allophycocyanin (APC) (Invitrogen, Carlsbad, CA) and/or anti-CD16-R-Phycoerythrin (PE) (Biolegend, San Diego, CA) in flow cytometry staining (FACS) buffer [HBSS with cations (Thermo Fisher Scientific), 0.2% human serum albumin (Grifols, Los Angeles, CA), 0.2% NaN_3_]. Approximately 10,000 events per sample were collected using an Accuri C6+ flow cytometer (BD Biosciences) and the percentage of CD15+/CD16+ cells was quantified using Accuri C6+ software (BD Biosciences). This method routinely yielded >99% neutrophil purity.

### Infection of Neutrophils With *F. tularensis* LVS

Neutrophils were resuspended in HBSS without divalent cations (Thermo Fisher Scientific) for enumeration and diluted to 2x10^7^/ml. Unless otherwise stated, neutrophils (5x10^6^/ml) were cultured in suspension (1-2 ml) in serum-free HEPES-buffered RPMI-1640 containing L-glutamine and phenol red (Lonza, Walkersville, MD). For lactate supplementation experiments, 4.7 mM sodium lactate (Sigma-Aldrich) was added to serum-free Hepes-buffered RPMI-1640 at time zero. For pyruvate feeding experiments, neutrophils (5x10^6^/ml) were cultured in suspension (1 ml) in serum-free HEPES-buffered SILAC RPMI 1640 Flex Media (Thermo Fisher Scientific) without glucose or glutamine and supplemented with 2 g/L sodium pyruvate (Sigma-Aldrich, Burlington, MA). Cultures were incubated in 14 ml polypropylene tubes at 37°C with 5% CO_2_ in the absence or presence of *F. tularensis* LVS as we previously described (24, 34). All experimental replicates were generated using neutrophils from at least three different donors.

### RNA Isolation and qRT-PCR

Total RNA was isolated from ultrapurified neutrophils at the indicated times using a Qiagen RNeasy kit (Hilden, Germany) according to the manufacturer’s instructions. RNA concentrations were measured by Nanodrop ND-1000 spectrophotometry. In an Eppendorf Mastercycler pro (Hamburg, Germany), RNA was reverse transcribed using the Invitrogen Super Script III First Strand Kit and cDNA was then amplified with gene-specific primer pairs (Origene, Rockville, MD) (primer sequences are in **Supplementary Table 1**) using Quanta Biosciences Perfecta SYBR Green Fast Mix (Gaithersburg, MD), all according to the manufacturer’s instructions. Melting curve analysis was used to check product specificity. The relative expression level of each transcript was determined using the 2^-ΔΔCt^ method and normalized to β-actin.

### Measurement of the Extracellular Acidification Rate Using Seahorse Analysis

Neutrophils were resuspended in XF assay media (Agilent Technologies, Santa Clara, CA) at a concentration of 1x10^7^/ml and 5x10^6^ cells per condition were plated onto a XF24 cell plate (Agilent Technologies) pre-coated with 0.1 mg/ml poly-L-lysine (Sigma-Aldrich). Plates were incubated at 37°C for 1 hr in the absence of CO_2_. ECAR was measured at 8.6-min intervals over a period of 95 min using a Seahorse XF24 analyzer and the Glycolysis Stress Test Kit (both from Agilent Technologies) according to manufacturer’s instructions. All data were analyzed using Seahorse Wave software (Agilent Technologies).

### Measurements of Lactate and Pyruvate

For measurements of lactate, supernatants (1 ml) from neutrophil cultures (1x10^7^/condition) were deproteinated by adding 1 ml ice-cold 0.5 M metaphosphoric acid, vortexing and placing on ice for 5 min. Supernatants were centrifuged at 10,000 x g at 4°C for 5 min to pellet proteins. Deproteinated supernatants were transferred to tubes containing 100 µl potassium carbonate to neutralize the acid and centrifuged at 10,000 x g at 4°C for 5 min to remove any precipitated salts. Samples for measurement of pyruvate (5x10^6^ cells/condition) were processed in this same manner. Lactate and pyruvate concentrations were measured using luminescence assay kits from Cayman Chemical (Ann Arbor, MI) according to the manufacturer’s instructions.

### Measurement of Intracellular ATP

Neutrophils (5x10^5^/condition) were transferred directly from culture tubes into a black 96-well plate and ATP concentrations were measured using a luminescence assay kit (Perkin Elmer, Waltham, MA) according to the manufacturer’s instructions.

### Gas Chromatography-Mass Spectrometry Metabolite Analysis

At indicated timepoints, ultrapure neutrophils (2.5x10^6^/condition) were washed with 1 ml HBSS without cations and pelleted at 1,000 x g for 5 min. Pellets were snap frozen and stored at -80°C. Data were obtained using a Trace 1310 Gas Chromatograph (Thermo Fisher Scientific) coupled with an ISQ LT Singe Quadrupole mass spectrometer (Thermo Fisher Scientific) and Xcalibur Software (Thermo Fisher Scientific). Metabolite peaks were detected using TraceFinder General Quant (Thermo Fisher Scientific) and metabolites were identified using a library of standards developed by the University of Iowa Metabolomics core facility. Metabolomics data were normalized to total ion signal and analyzed by MetaboAnalyst (http://www.metaboanalyst.ca).

### Quantitation of Apoptosis

At the indicated time points, apoptosis was measured by flow cytometric analysis of phosphatidylserine (PS) externalization using Annexin V-FITC, with addition of propidium iodide (PI) to detect plasma membrane permeabilization and progression to late apoptosis/secondary necrosis as we described (23, 34). In brief, neutrophils (5x10^5^/condition) were costained with Annexin V-FITC and PI (both from BioVision, Milpitas, CA) in binding buffer (10 mM HEPES pH 7.4, 140 mM NaCl, 2.5 mM CaCl_2_) for 5 min, in the dark. Approximately 10,000 events per sample were collected using an Accuri C6+ flow cytometer and the data were analyzed using Accuri C6+ software.

### Inhibitor Treatments

Inhibitors were added to neutrophils at the following final concentrations 1 hr prior to infection, unless otherwise indicated: 20 µM WZB-117 (20 mM stock in DMSO, Sigma-Aldrich), 5 mM 2-deoxy-D-glucose (2-DG) (100 mM stock in RPMI-1640, Sigma-Aldrich), 50-100 μM 3-(3-pyridinyl)-1-(4-pyridinyl)-2-propen-1-one (3PO, 100 mM stock in DMSO, Sigma-Aldrich), 20 µM CP-91149 (20 mM stock in DMSO, Sigma-Aldrich), or 40 µg/ml chlorogenic acid (40 mg/ml stock in DMSO, Sigma-Aldrich). Q-VD-OPh (10 mM stock in DMSO, Cayman Chemical) was added to a final concentration of 10 µM, at time zero simultaneously with initiation of infection (no pretreatment).

### Immunoblotting

Neutrophils were lysed with 10% NP-40 in Tris-buffered saline supplemented with protease and phosphatase inhibitors (36.76 µg/ml Aprotinin, 43.2 mM Levamisole, 8.65 mM AEBSF, 40.5 µg/ml Leupeptin, 1.76 mM PMSF, 13.2 nM Pepstatin A, 6.76X Protease inhibitor cocktail (all Sigma-Aldrich) and 6.76x phosphatase inhibitor cocktail (Thermo Fisher Scientific). Proteins were separated on NuPAGE 4–12% Bis-Tris gradient gels (Invitrogen) and transferred to polyvinylidene difluoride membranes (Perkin Elmer). Membranes were blocked with 5% bovine serum albumin in Tris-buffered saline with 0.1% Tween 20, then probed with 1:500 rabbit anti-XIAP (23453-1-AP, Proteintech, Rosemont, IL), 1:300 rabbit anti-MCL-1 (16225-1-AP, Proteintech). 1:500 mouse anti-caspase-3 (clone C33, 3004, BioVision), 1:500 rabbit anti-GBE1 (HPA038073, Sigma-Aldrich), 1:500 rabbit anti-UGP2 (HPA034697, Sigma-Aldrich), or 1:500 rabbit anti-GYS1 (3893S, Cell Signaling Technologies, Danvers, MA). The anti-β-actin (NB600-503SS, Novus Biologicals, Littleton, CO) loading control was used at 1:2,000. Bands were detected using 1:2000 horseradish‐peroxidase‐conjugated secondary antibodies (Cytiva, Marlborough, MA) and the Pierce SuperSignal West Femto chemiluminescent substrate (Thermo Fisher Scientific) and the Odyssey Fc imaging system (LI‐COR Biosciences, Lincoln, NE).

### Quantitation of Glucose Uptake

Glucose uptake was measured at the indicated time points using the zero trans method, as previously described (35). Specifically, neutrophils (2x10^5^/condition) were centrifuged at 1,400 x g for 5 min, resuspended in glucose-free RPMI-1640 and incubated at 37°C with 5% CO_2_ for 5 min. [^3^H]2-deoxy-D-glucose (1 μCi/ml) was added to a final concentration of 100 μM (0.1 μCi/ml, 1 μCi/sample) and samples were incubated at 37°C for 3 min. Ice-cold glucose free-RPMl 1640 containing 0.3 mM phloretin (Cayman Chemical) was added and samples were placed on ice for 5 min to stop glucose uptake. Samples were centrifuged through a 50 µl cushion of ice-cold 10% (w/v) bovine serum albumin at 8,800 x g for 30 sec and cells were lysed in 100 µl of 1% Triton X-100. Lysates were transferred to scintillation vials along with 5 ml Econo-Safe Economical Biodegradable Counting Cocktail (Research Products International, Mount Prospect, IL) and vials were shaken for 10 sec before counting in a LS6500 Multi-Purpose Liquid Scintillation Counter (Beckman Coulter, Brea, CA).

### Measurement of Intracellular Glycogen Stores

Neutrophils (1×10^6^/condition) were lysed with 200 μl ice-cold ddH_2_O and boiled at 95°C for 10 min. Lysates were centrifuged at 18,000 x g at 4°C for 10 min to remove insoluble material. Glycogen concentrations were measured by colorimetric assay, according to kit instructions (BioVision).

### Statistical Analyses

All data are plotted as mean ± SEM and represent at least three independent experiments. Data were analyzed using GraphPad Prism version 8 or 9 with p < 0.05 dictating statistical significance. Data from experiments with one variable were analyzed *via* Student’s *t*-test. Data from experiments with multiple variables were analyzed *via* two-way ANOVA and Tukey’s multiple-comparisons posttest. Additional details are provided in the figure legends.

## Results

### Genes Encoding Glycolytic Enzymes and Glucose Transporters Are Upregulated by LVS Infection

**Figure 1:**
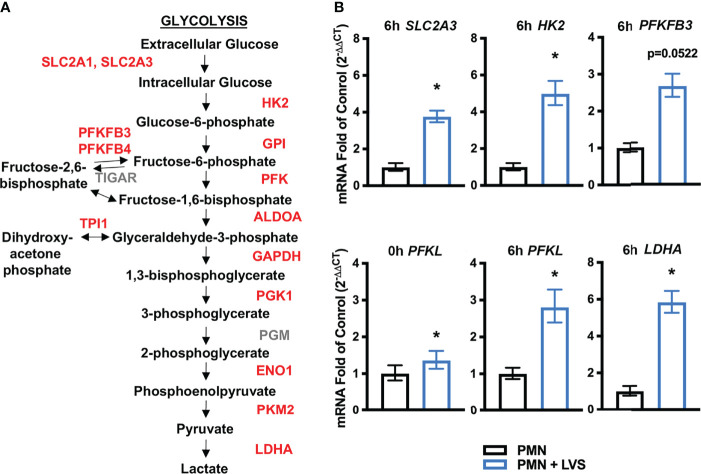
LVS significantly upregulates PMN genes encoding glycolytic enzymes and glucose transporters. **(A)** Glycolysis pathway genes shown in red are significantly upregulated relative to uninfected cells by microarray analysis (22). **(B)** Validation of the microarray data by qRT-PCR analysis of *SLC2A3* (6 hpi), *HK2* (6 hpi), *PFKFB3* (6 hpi), *PFKL* (0 hpi and 6 hpi), and *LDHA* (6 hpi), n=3, *p < 0.05.

We demonstrated previously that both type B (LVS) and type A (Schu S4) *F. tularensis* strains significantly delay the onset of apoptosis as a means to extend human neutrophil lifespan and this is achieved, at least in part, by transcriptional reprogramming (22–24). Our prior analysis of the microarray dataset focused on differential expression of genes encoding apoptosis regulators and cell survival factors. However, these data also revealed significant differential expression of ~800 genes linked to metabolism, and glycolysis was among the top pathways identified by KEGG analysis at all times points examined 3-24 hours post infection (hpi) (22). Specifically, 10 of 11 genes encoding glycolysis enzymes were induced by LVS along with the two main glucose transporters (GLUTs) that human neutrophils express (**Figure 1A**) (22, 36).

Herein, we validated the microarray data using qRT-PCR and our data demonstrate that expression of genes encoding hexokinase-2 (*HK2*), lactate dehydrogenase (*LDHA*) and GLUT-3 (*SLC2A3*) were significantly increased by 6 hpi (**Figure 1B**). *PFKFB3* (6-phosphofructo-2-kinase/fructose-2,6-bisphosphatase) expression followed a similar trend of upregulation but did not reach statistical significance (**Figure 1B**). Phosphofructokinase (*PFKL*) expression was significantly higher in LVS-infected neutrophils immediately following infection and at 6 hpi (**Figure 1B**). Thus, our current and published data demonstrate upregulation of genes linked to glycolysis during LVS infection.

### LVS Infection Increases Glycolysis and Glycolytic Capacity

**Figure 2:**
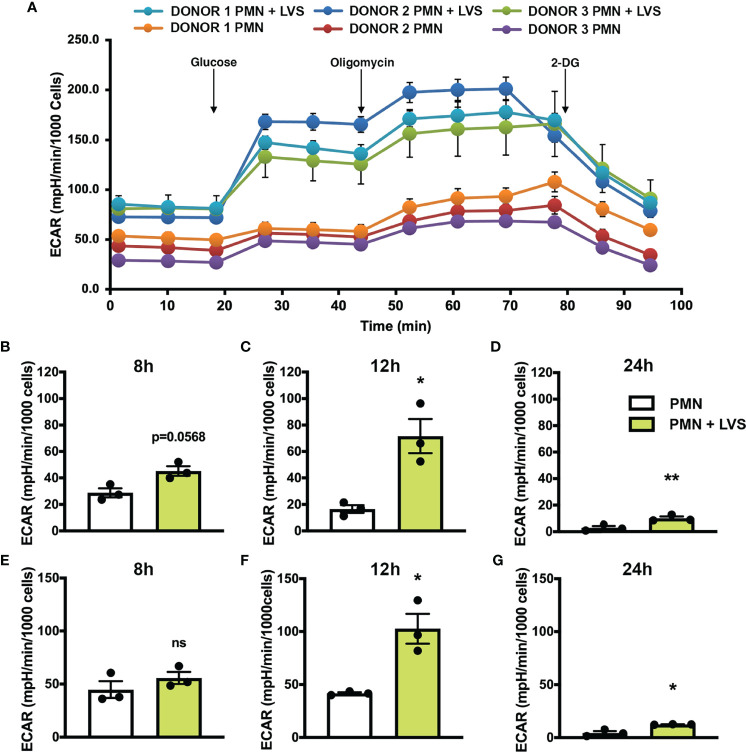
LVS infection upregulates PMN glycolysis. Glycolytic function measured by Seahorse analysis as extracellular acidification rate (ECAR) in control and LVS-infected PMNs, n=3. **(A)** ECAR curves at 12 hr. Glucose was injected to a final concentration of 10 mM at 20.84 min, oligomycin was injected to a final concentration of 1 µM at 46.78 min and 2-DG was injected to a final concentration of 50 mM at 72.83 min. Where not visible, error bars are smaller than symbols. Quantitation of glycolysis **(B–D)** and glycolytic capacity **(E–G)** in control and LVS-infected PMNs at 8, 12 and 24 hpi. *p < 0.05. **p < 0.01. ns, not significant.

Based on our gene expression data, we hypothesized that glycolytic activity was increased in LVS-infected neutrophils. To address this, we used a Seahorse metabolic analyzer to measure glycolysis and glycolytic capacity in neutrophils at various timepoints after infection with comparison to the uninfected controls (**Figure 2**). Seahorse curves obtained at 12 hr are shown in **Figure 2A**. Pooled data show that in agreement with our microarray data, glycolysis (**Figures 2B–D**) and glycolytic capacity (**Figures 2E–G**) of LVS-infected neutrophils were significantly increased by 12 hpi (**Figures 2C, F**) and were sustained thereafter, although overall responses for both infected and control cells were lower at 24 hr (**Figures 2D, G**).

**Figure 3:**
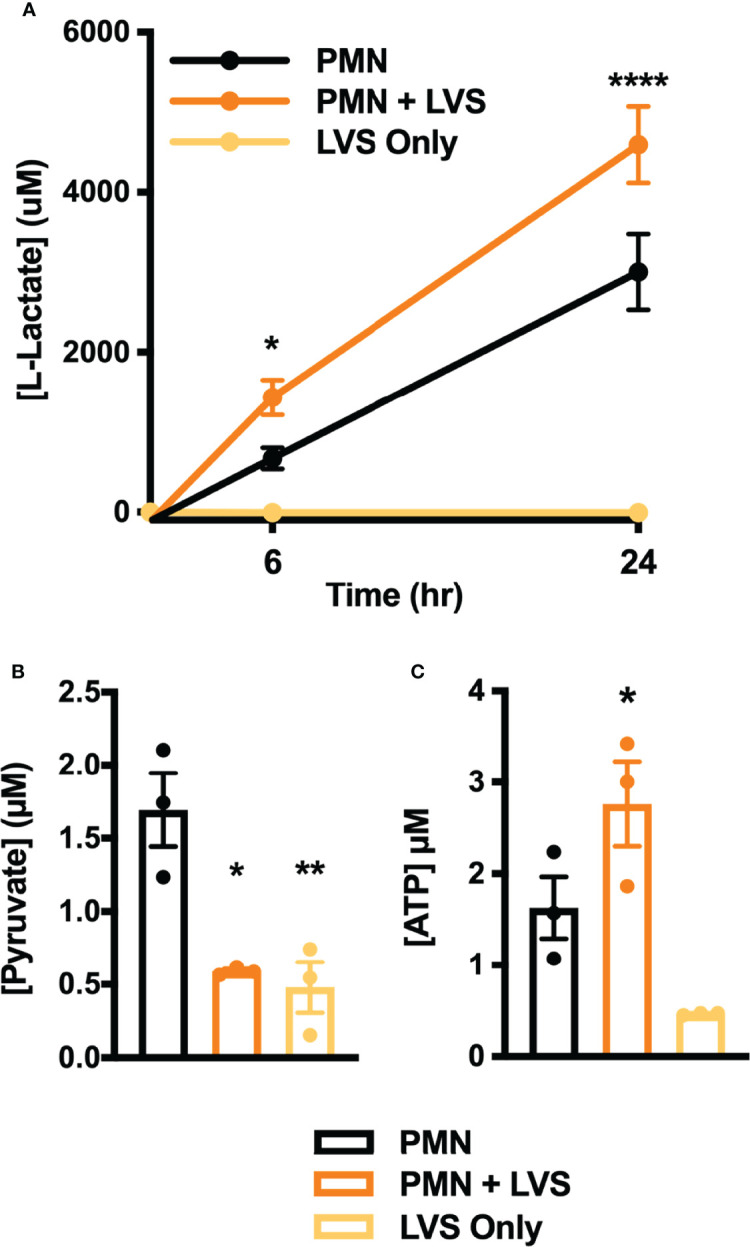
Differential effects of LVS on PMN lactate, ATP, and pyruvate. **(A)** Amount of lactate released by control and infected PMNs or LVS alone at 0, 6 and 24 hpi, n=3-4. ****p < 0.0001, *p < 0.05 vs. uninfected control cells at the indicated time points. **(B, C)** Pyruvate **(B)** and ATP **(C)** levels of control and infected PMNs or LVS alone at 24 hr, n=3. *p < 0.05, **p < 0.01 vs. control PMNs as indicated.

**Figure 4:**
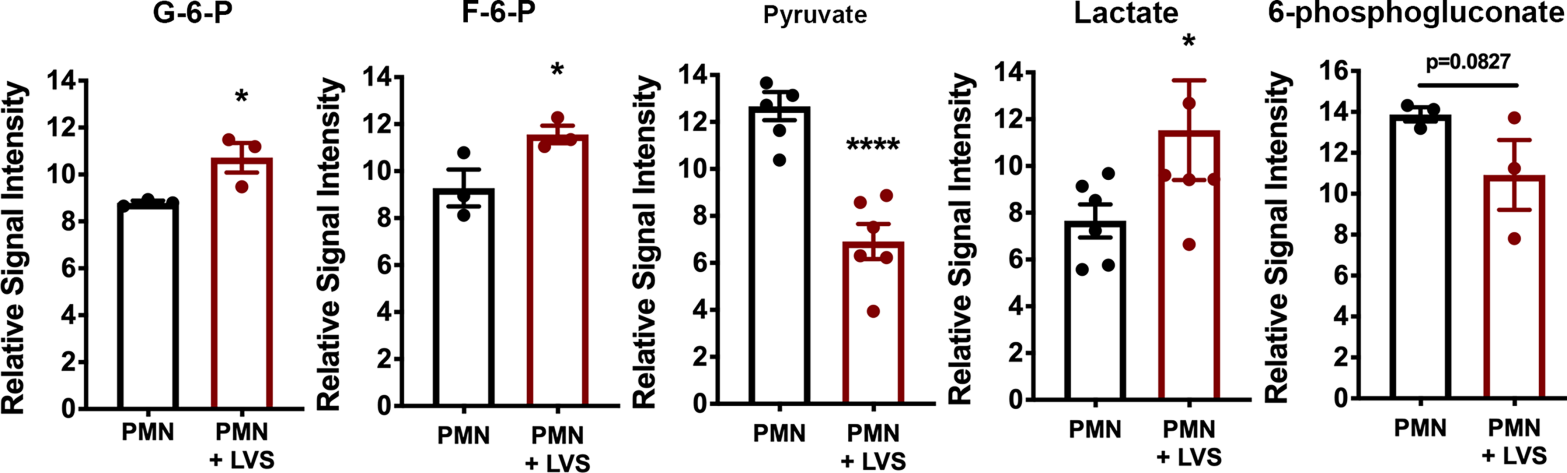
Differential effects of LVS infection on PMN metabolite levels. The signal intensities of glucose-6-phosphate (G-6-P), fructose-6-phosphate (F-6-P), pyruvate, lactate and 6-phosphogluconate, relative to total ion signal, measured by GC-MS in control and LVS-infected PMNs at 9 hpi, n=3-6. *p < 0.05, ****p < 0.0001 as indicated.

A key outcome of glycolysis is lactate secretion, which drives ECAR detected by Seahorse analysis. Congruent with the data shown in **Figure 2**, lactate progressively increased in neutrophil extracellular medium and was significantly more abundant following LVS infection than the uninfected controls (**Figure 3A**). Conversely, extracellular pyruvate levels were significantly reduced (**Figure 3B**). In addition, LVS-infected neutrophils contained significantly more intracellular lactate (*p* < 0.001) and a significantly higher ratio of lactate to pyruvate than control neutrophils (*p* < 0.01) at all timepoints examined (**Supplementary Figure 1**). In agreement with the fact that mature human neutrophils rely primarily on glycolysis for ATP generation (6), we also show that LVS-infected neutrophils contained significantly more ATP than their uninfected counterparts at 24 hpi (**Figure 3C**). Bacterial lactate, pyruvate, and ATP were also quantified (**Figures 3A–C**), and these data demonstrate that LVS metabolites cannot account for the differences between control and infected neutrophils. Significant induction of glycolysis was independently confirmed by quantitation of metabolites using GC-MS (**Figure 4**). These data confirm the differential abundance of lactate and pyruvate shown in **Figure 3** and extend these data to demonstrate that fructose-6-phosphate and glucose-6-phosphate were also more abundant in the infected PMNs by 9 hpi. In contrast, levels of the TCA cycle intermediates succinate and citrate were unchanged (data not shown).

KEGG analysis of our microarray data also identified the pentose phosphate pathway (PPP)/hexose monophosphate shunt (HMS), but in this case expression of genes encoding pathway enzymes was significantly downregulated rather than induced between 6 and 24 hpi, including glucose-6-phosphate dehydrogenase (*G6PDH*), 6-phosphogluconate dehydrogenase (*PGD*), 6-phosphogluconolactonase (*6PGLS*) and ribose-5-phosphate epimerase (*RPIA*) (22). In agreement with this, 6-phosphogluconate was slightly less abundant in neutrophils after LVS infection by GC-MS (**Figure 4**).

Taken together, our biochemical analyses demonstrate significant induction of glycolysis and glycolytic capacity in LVS-infected PMNs leading to enhanced ATP production, lactate release and extracellular acidification that was not coupled to induction of the PPP.

### Glycolysis Inhibition Blocks the Ability of LVS to Delay PMN Apoptosis

**Figure 5:**
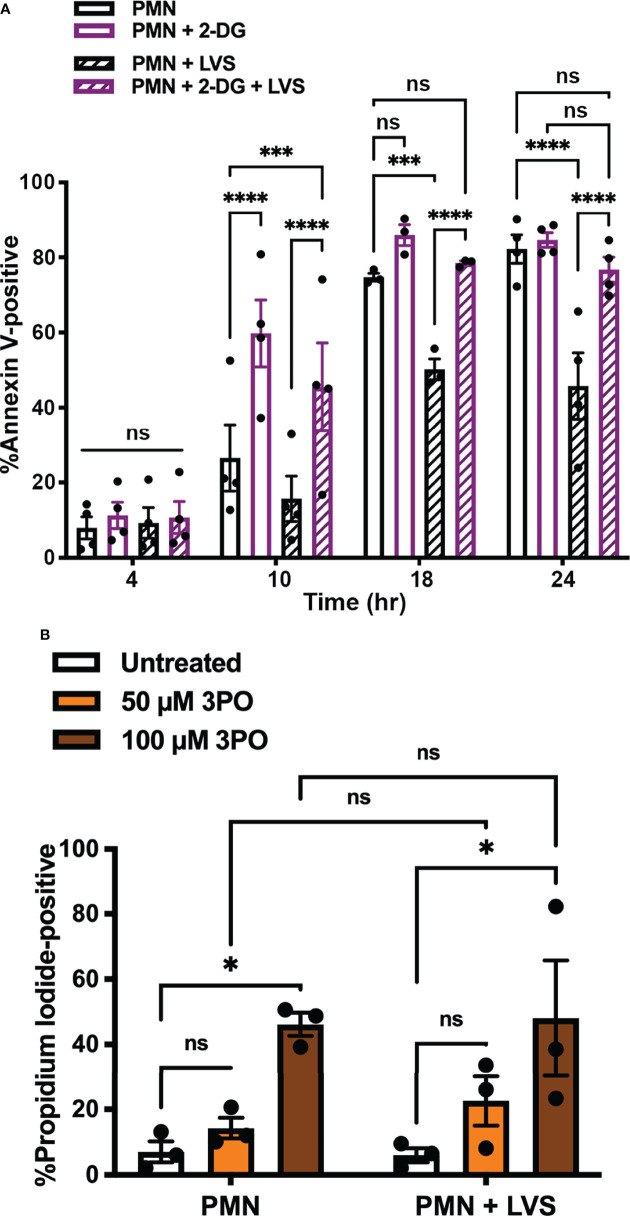
Glycolysis inhibition blocks the ability of LVS to delay PMN apoptosis. **(A)** Effects of 2-DG on the kinetics of apoptosis of control and LVS-infected cells were measured using Annexin V-FITC staining and flow cytometry at the indicated time points, n=4. ***p < 0.001, ****p < 0.0001, ns, not significant, as indicated. **(B)** Effects of 3PO on PMN viability at 24 hr as measured by PI staining and flow cytometry, n=3. *p<0.05, ns, not significant, as indicated.

Our next objective was to determine if glycolysis induction contributed to the ability of *F. tularensis* to extend neutrophil lifespan. To interrogate this potential link, we inhibited hexokinase by treatment with the glucose analog, 2-deoxy-D-glucose (2-DG) (37), and used our established methods to quantify the kinetics of PMN apoptosis using Annexin V-FITC/PI co-staining and flow cytometry (**Figure 5A**) (24, 34). Consistent with our published data, significantly fewer LVS-infected PMNs were apoptotic at 24 hpi as compared with the uninfected controls (45.75 ± 8.9% vs. 82.2 ± 3.8%, *p* < 0.0001, n=4). However, inhibition of PMN glycolysis with 2-DG prior to infection ablated the ability of LVS to extend neutrophil lifespan and at the same time accelerated constitutive apoptosis of uninfected PMNs as indicated by detection of externalized PS by Annexin V-FITC staining at 10 hr (*p* < 0.0001) (**Figure 5A**). Similarly, the PFK and PFKFB3-inhibitor 3PO (38) undermined PMN survival in a dose-dependent manner and increased the percentage of cells that were PI-positive at 24 hr, indicating progression from early apoptosis to late apoptosis/secondary necrosis (24). Pooled data are shown in **Figure 5B**, and representative flow cytometry dot plots are shown in **Supplementary Figure 2**. Based on these data, we conclude that glycolysis was essential for PMN survival and the ability of LVS to delay apoptosis.

### Pyruvate Preferentially Accelerates Death of LVS-Infected Neutrophils

As neutrophils have the capacity for gluconeogenesis and can convert pyruvate into glucose-6-phosphate (26), we tested the ability of pyruvate to support PMN survival. To this end, cells were cultured in either in normal RPMI-1640 (which contains 2 g/L glucose) or in medium where glucose was replaced with 2 g/L pyruvate. Under these conditions, apoptosis of LVS- infected PMNs was significantly increased by 6 hpi and glycogen stores and lactate production were significantly diminished (**Supplementary Figures 3A–C**). At this same time point, lactate production by uninfected PMN was also significantly reduced, but glycogen stores and apoptosis were unchanged relative to the glucose-fed controls. These data suggest that pyruvate can sustain viability of uninfected PMNs for at least 6 hr. By 24 hr, nearly all pyruvate fed cells were Annexin V-positive and ATP depleted (**Supplementary Figures 3A, D**). Moreover, a majority of cells had progressed to late apoptosis/secondary necrosis by 24 hr, but ~80% of infected PMNs were PI-positive as compared with only ~60% of their uninfected counterparts (*p*<0.0001) (**Supplementary Figure 3E**). These data identify additional differences between uninfected and infected PMNs and further support the hypothesis that glycolysis was vital for neutrophil survival and extended lifespan after LVS infection.

### Glucose Uptake Is Also Increased and Required For Delayed Apoptosis

**Figure 6:**
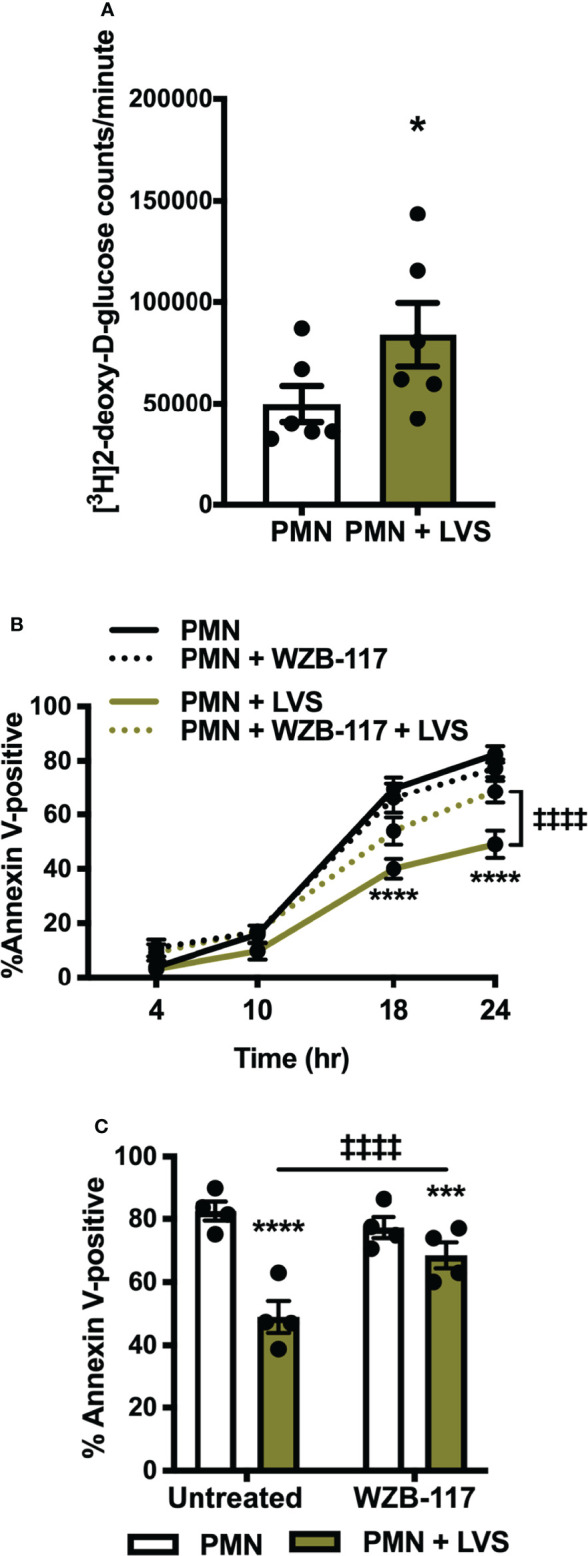
Glucose uptake is increased in LVS-infected PMNs and is required for apoptosis delay. **(A)** [^3^H]2-deoxy-D-glucose uptake was measured at 12 hr. *p < 0.05, n=6. **(B, C)** Apoptosis of control and LVS-infected PMNs in the presence or absence of WZB-117 measured by Annexin V-FITC staining and flow cytometry at the indicated timepoints **(B)** and at 24 h **(C)**. *p < 0.05, ***p < 0.001, ****p < 0.0001 compared to untreated, control PMNs, ^‡‡‡‡^ *p* < 0.0001 compared to untreated, LVS-infected PMNs as indicated, n=4.

To understand what could be linking apoptosis and glycolysis, we sought to determine what fueled the glycolytic upregulation in LVS-infected neutrophils. As glucose is the substrate of glycolysis, and expression of the genes encoding GLUT-1 (*SLC2A1*) and GLUT-3 (*SLC2A3*) were significantly increased after infection (**Figures 1A, B**) (22), we hypothesized that glucose uptake may also be enhanced. To test this, we used a sensitive and quantitative radiolabeled glucose uptake assay (35), and the data in **Figure 6A** indicate that by 12 hpi, transport of glucose into LVS-infected PMNs was significantly increased relative to the uninfected controls.

WBZ-117 inhibits all glucose transporter isoforms that are expressed in PMNs [GLUT-1, GLUT-3 and GLUT-4 (39)] and pretreatment of PMNs with WBZ-117 significantly diminished the ability of LVS to sustain PMN viability at 18 and 24 hpi, but unlike 2-DG (**Figure 5A**) did not accelerate constitutive apoptosis of the uninfected controls. Pooled data are shown in **Figures 6B, C** and representative flow cytometry dot plots are shown in **Supplementary Figure 4**. Neutrophils can also store glucose-6-phosphate in the endoplasmic reticulum for later use, but we detected no effects on apoptosis when we treated cells with the glucose-6-phosphate translocase (G6PT) inhibitor, chlorogenic acid (40) (**Supplementary Figure 5**). These data demonstrate that glucose uptake was significantly increased by LVS and was essential for delayed apoptosis of PMN after infection, whereas glucose-6-phosphate storage in the endoplasmic reticulum did not appear to play a role.

### Glycogen Dynamics Are Complex and Differ in Control and Infected Neutrophils

**Figure 7:**
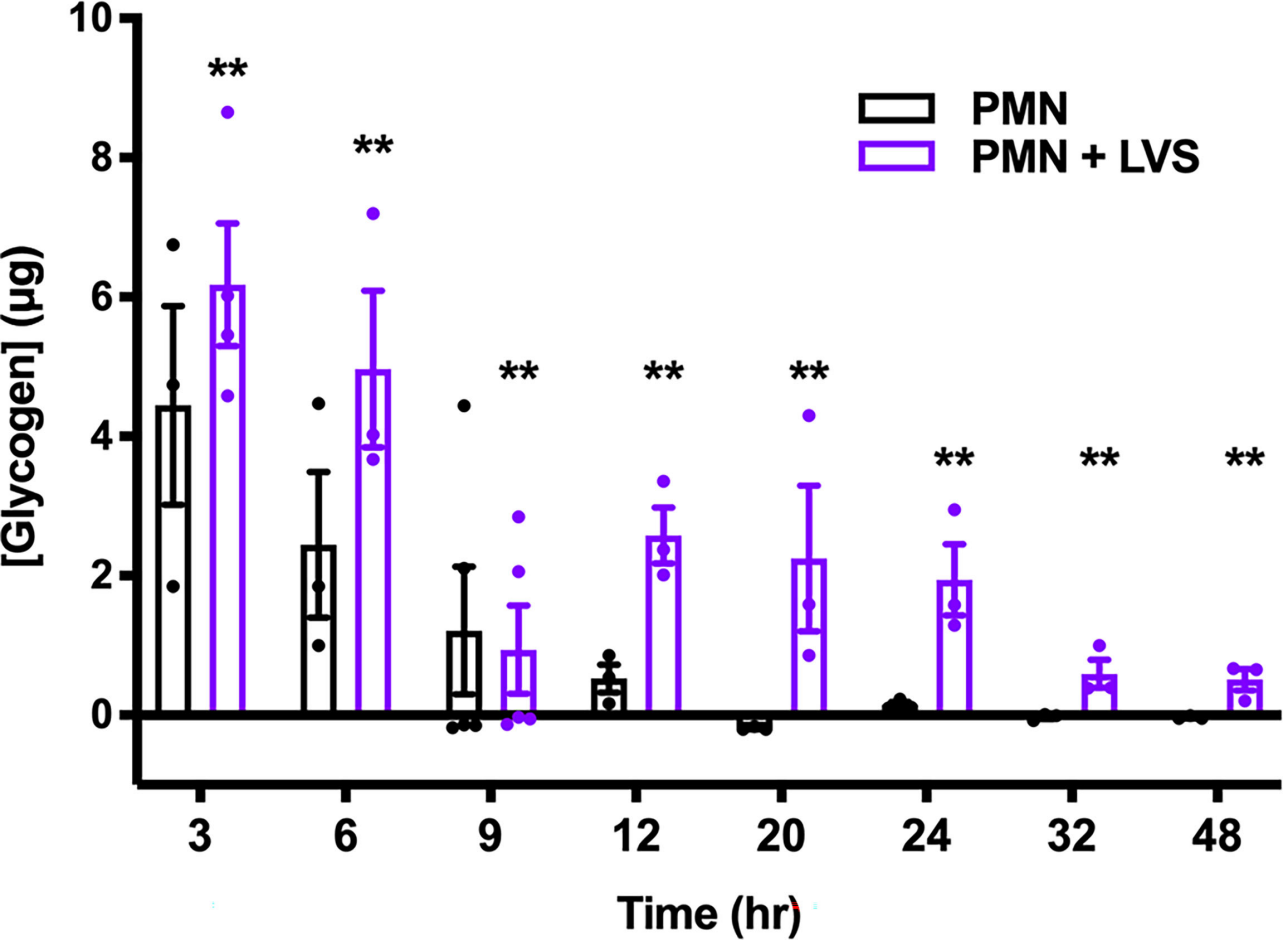
LVS modulates PMN glycogen dynamics and abundance. Glycogen stores of control and LVS-infected PMNs were quantified at the indicated timepoints, n=3-5. **p < 0.01 compared to control PMNs at each timepoint.

To ascertain the role of glycogen as a candidate regulator of PMN lifespan, we first quantified glycogen levels at multiple time points over 48 hr and the data shown in **Figure 7** are noteworthy for several reasons. First, we predicted that LVS-infected neutrophils would consume more glycogen to fuel increased glycolysis, and that glycogen levels would therefore be lower in these cells, but to our surprise LVS-infected neutrophils contained significantly more glycogen than the controls at nearly every assayed timepoint. Second, we show that glycogen was most abundant in all PMNs at 3 hr, the earliest time point examined. Thereafter, glycogen stores of the control PMNs declined progressively. In marked contrast, glycogen stores of LVS-infected PMNs reached a nadir at 9 hpi but were then replenished. Thus, at 12-24 hpi, glycogen stores of LVS-infected PMNs were similar to the control cells at 6 hr and declined significantly only at 32 and 48 hpi. Taken together, these data demonstrate that glycogen stores are dynamically regulated, and that this storage form of glucose was significantly more abundant in LVS-infected PMNs at both early and later stages of infection. Nonetheless, genes encoding the three main enzymes of glycogenesis, UDP-glucose pyrophosphorylase 2 (*UGP2*), glycogen branching enzyme (*GBE1*) and glycogen synthase (*GYS1*) were not differentially expressed as judged by qRT-PCR (**Supplementary Figure 6A**) and LVS also had no apparent effect on the levels of each enzyme as judged by immunoblotting of cell lysates (**Supplementary Figure 6B**). Thus, the ability of LVS-infected PMNs to replenish glycogen stores at 12 hpi cannot be attributed to changes in the abundance of biosynthetic pathway enzymes.

**Figure 8:**
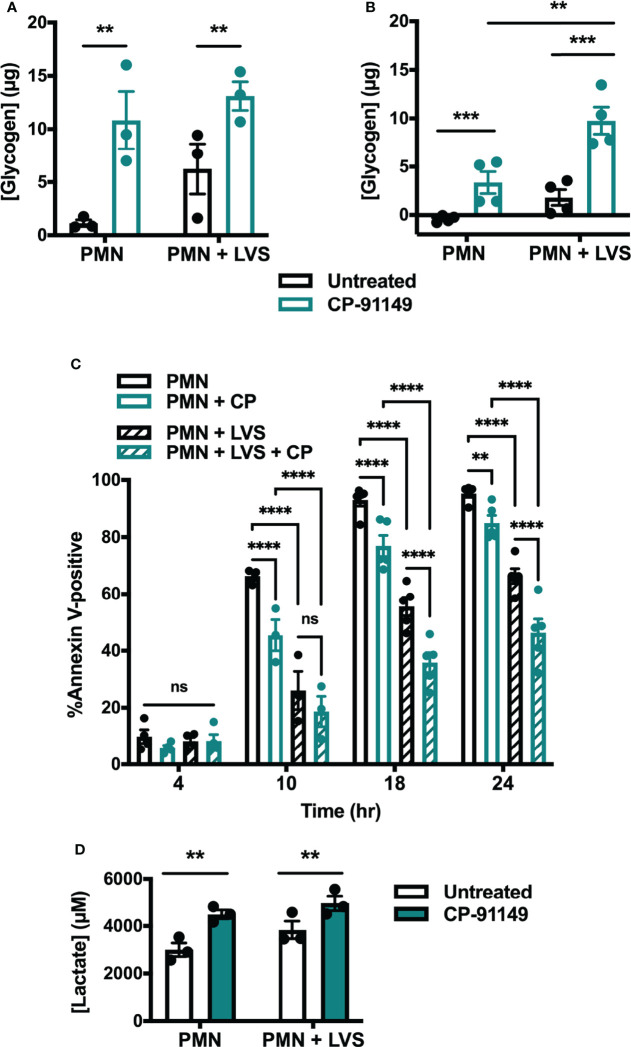
Glycogen abundance correlates with delayed apoptosis. **(A, B)** Glycogen abundance in uninfected and LVS-infected PMNs in the presence or absence of CP-91149 at 6 hr **(A)** and 24 hr **(B)**, n=3-4. **p < 0.01, ***p < 0.001 as indicated. **(C)** Apoptosis of uninfected and LVS-infected PMNs in the presence or absence of CP-91149 quantified using Annexin V-FITC staining and flow cytometry at the indicated timepoints, n=3-5. **p < 0.01, ****p < 0.0001, ns, not significant, as indicated. **(D)** Amount of lactate released by control and LVS-infected PMNs in the presence or absence of CP-91149 at 12 hr, n=3. **p < 0.01 as indicated.

To demonstrate a definitive link between glycogen abundance and cell longevity we assessed the extent to which pharmacologically increasing glycogen affected apoptosis. To this end, we treated cells with CP-91149, a glycogen phosphorylase inhibitor that effectively prevents glycogen breakdown (41). Following CP-91149 treatment we quantified glycogen levels, apoptosis, and lactate release (**Figure 8**). As expected, neutrophils treated with CP-91149 contained significantly more glycogen at 6 hr (**Figure 8A**) and 24 hr (**Figure 8B**) than their untreated counterparts. Specifically, CP-91149 increased glycogen levels ~10-fold at 6 hr in the uninfected PMNs and ~2-fold in the LVS-infected PMNs. At the same time, CP-91149 treatment significantly delayed apoptosis of both control and LVS-infected PMNs as indicated by Annexin V-FITC staining (**Figure 8C**), and significantly increased glycolysis as indicated by quantitation of lactate release (**Figure 8D**). We therefore conclude that inhibition of glycogen catabolism was sufficient to delay constitutive apoptosis and increase glycolysis of uninfected control PMNs and further enhanced the ability of LVS to extend PMN lifespan and upregulate glycolysis.

### Caspase Inhibition Increases PMN Glycolysis, Glycolytic Capacity, and Glycogen Stores

**Figure 9:**
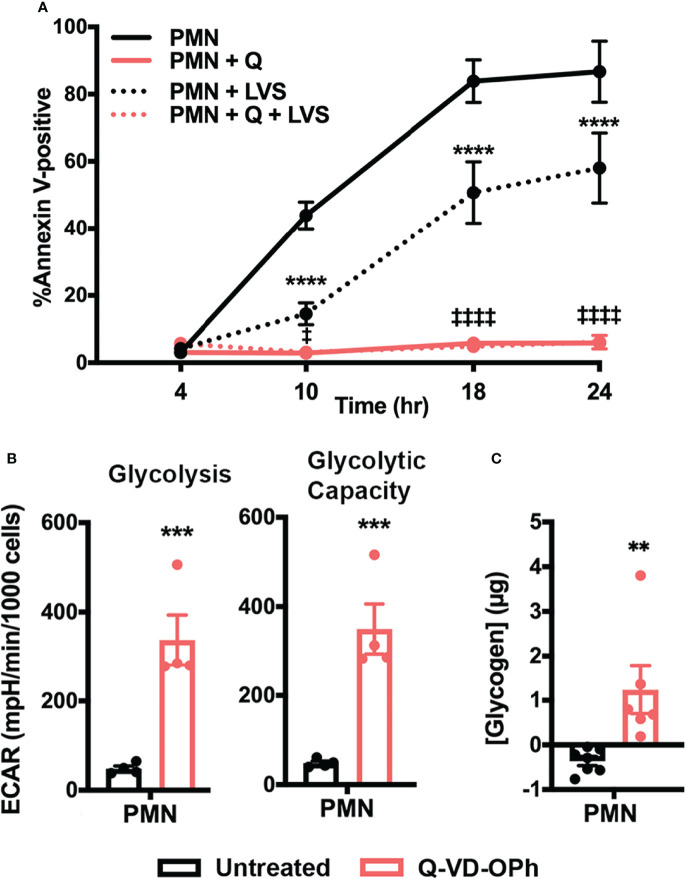
Caspase inhibition increases PMN glycolysis, glycolytic capacity, and glycogen abundance. **(A)** Kinetics of apoptosis of control and Q-VD-OPh-treated PMNs were measured using Annexin V-FITC staining and flow cytometry. Data are the mean ± SEM, n=3. ****p < 0.0001 compared to untreated, uninfected PMNs, ^‡^ *p* < 0.05 and ^‡‡‡‡^ *p* < 0.0001 compared to untreated, LVS-infected PMNs at the indicated time points. **(B)** Quantitation of glycolysis and glycolytic capacity measured by Seahorse analysis as extracellular acidification rate (ECAR) in PMNs in the presence or absence of Q-VD-OPh at 12 hr, n=4. ***p < 0.001. **(C)** Measurement of glycogen abundance in PMNs in the presence or absence of Q-VD-OPh at 24 hr, n=6-7. **p < 0.01.

To further examine the link between neutrophil metabolism and apoptosis, we asked if inhibiting caspases pharmacologically with the pan-caspase inhibitor Q-VD-OPh (42) would phenocopy the increased glycolysis and glycogen abundance observed in LVS-infected neutrophils. As shown in **Figure 9**, Q-VD-OPh treatment nearly ablated PMN apoptosis over the time course examined, as 94.13 ± 1.7% of drug-treated cells remained viable at 24 hr (**Figure 9A**). We next measured glycolytic function using Seahorse analysis and show that this pan-caspase inhibitor increased neutrophil glycolysis and glycolytic capacity ~7-fold at 12 hr (**Figure 9B**). Finally, we demonstrate that Q-VD-OPh-treated cells also contained significantly more glycogen at 24 hr (**Figure 9C**). These data further support the hypothesis that neutrophil survival and glycolysis are fundamentally linked and reinforce the notion that glycogen abundance correlates with delayed apoptosis.

### Effects of Lactate and 2-DG on Apoptosis and Apoptosis Regulatory Factors

Precisely how glycolysis is linked to PMN longevity in our system is unknown. Thus, we tested whether the amount of lactate secreted by LVS-infected PMNs (4.7 mM) was sufficient to alter constitutive apoptosis. Specifically, lactate was added to uninfected PMNs at time zero, and the rate of apoptosis was determined using Annexin V-FITC/PI staining over 24 hr. By this assay, lactate had no discernable effect on constitutive PMN death (**Supplementary Figure 7**).

To interrogate the effects of 2-DG, we used western blotting. As shown in **Supplementary Figure 8A**, 2-DG treatment increased processing of procaspase-3 to its cleaved, mature form in both uninfected and infected PMNs, confirming the flow cytometry data shown in **Figure 5A**. As XIAP plays a role in caspase-3 inhibition and is maintained at high level in LVS-infected PMNs (22), we predicted that levels of this anti-apoptosis regulatory factor would be diminished in cells treated with 2-DG. The data in **Supplementary Figure 8B** confirm disappearance of XIAP from control PMNs by 24 hr and its sustained abundance in cells infected with LVS, but to our surprise, 2-DG did not cause XIAP to disappear from infected PMNs. Similarly, 2-DG had no apparent effect on levels MCL-1 (**Supplementary Figure 8C**).

## Discussion

Neutrophils are key mediators of infection resolution that are inherently short-lived and undergo constitutive apoptosis approximately 24 hours after release into circulation (4). This tightly regulated lifespan is intimately linked to cell function, as disruption of neutrophil turnover can lead to exacerbated disease and tissue destruction (18, 20, 21). Published data from our group demonstrate that infection with *F. tularensis* strains, including LVS and Schu S4, significantly extends neutrophil lifespan (16, 22, 23), but the mechanisms enabling infected neutrophils to override this strictly regulated cell death program are not fully understood.

The realization that metabolism can directly regulate inflammation and immune cell function has significant implications for our understanding of the molecular mechanisms associated with control or exacerbation of infection as well as cancer, obesity, autoimmunity and atherosclerosis. A majority of studies in the rapidly advancing field of immunometabolism have focused on macrophages and T lymphocytes, and relatively few have included or focused on neutrophils or other leukocyte types (7, 25, 26, 28, 30, 31). The central finding of this study is that *F. tularensis* elicits a metabolic program in human neutrophils that is distinct from other stimuli described to date and is essential for cell longevity and delayed apoptosis.

Our published data establish that LVS infection significantly dysregulates over 800 neutrophil genes associated with metabolism, including upregulation of genes encoding nearly every glycolytic enzyme and the two main glucose transporters used by neutrophils (22). PFKL and HK2 are the two most prominent regulatory enzymes of glycolysis, and we confirm that in LVS-infected neutrophils *HK2* expression increased nearly 6-fold and *PFKL* expression increased ~3-fold by 6 hpi (**Figure 1**). Notably, significant induction of *PFKL* expression was apparent within minutes of LVS addition (0 hpi), reinforcing the notion that *F. tularensis* can act at a distance and begin to alter neutrophil function at the earliest stages of infection prior to bacterial binding and phagocytosis (43). At the biochemical level, changes in PMN gene expression led to increases in glucose uptake (**Figure 6A**) as well as glycolysis and glycolytic flux (**Figure 2**) that peaked at 12 hpi and were accompanied by accumulation of pathway intermediates as well as ATP and lactate (**Figures 3**, **4**; **Supplementary Figure 1**). The LDHA isoform of lactate dehydrogenase catalyzes the conversion of pyruvate to lactate (44), and following LVS infection, *LDHA* expression increased ~6-fold by 6 hpi (**Figure 1**). Furthermore, increases in lactate and the lactate/pyruvate ratio and reduction of pyruvate were apparent by GC-MS as early as 3 hpi and were sustained to at least 24 hpi (**Supplementary Figure 1**; **Figures 3B**, **4**), and lactate also accumulated in the extracellular medium (**Figure 3A**). The opposing effects of LVS on lactate and pyruvate abundance contrasts with simultaneous increases in both metabolites that typically accompany PMN activation and microbe killing, and it has been proposed that changes in the ratio of these metabolites may be a hallmark of metabolic reprogramming (27, 32).

PMNs store glucose as glycogen, a branched glucose polymer that surrounds a glycogenin core. ^14^C-glucose pulse-chase experiments demonstrated that glycogen stores are highly dynamic and are constantly adding and removing glucose units even at steady state (45). Accordingly, glycogenolysis ensues immediately in glucose free medium, reducing stores by up to 38% in 60 min, and glycogen is rapidly resynthesized when exogenous glucose is restored. As little as 5.3 μg glucose/10^6^ cells/60 min is sufficient to maintain glycogen stores and 74 μg/10^6^ cells supports maximum resynthesis (45). Enzymes that mediate glycogen synthesis and their encoding mRNAs are long-lived, and both glycogen synthase and glycogen phosphorylase bind glycogen (45). It has long been believed that neutrophils use glycogen for fuel only during phagocytosis and when extracellular glucose is scarce (6, 7, 46). However, recent reports (26) and the results of this study (**Figure 7**) contest this model. We show here that glycogen levels in uninfected neutrophils declined progressively as cells aged despite being cultured in glucose-replete medium. On the other hand, glycogen dynamics of LVS-infected cells were characterized by waves of net glycogenolysis and glycogenesis. Glycogen stores were highest at 3 hr and were more abundant in LVS-infected cells than the uninfected controls. Although glycogen stores declined to a nadir at 9 hpi, they were replenished in the infected cells by 12 hpi and then maintained at a distinct intermediate level, before declining again at 32-48 hpi. To our knowledge, changes in glycogen stores of aging PMNs have not previously been reported. As near total glycogen depletion in control PMNs at 9-12 hr coincides with the onset of caspase-3 activation and progression to apoptosis (**Figures 7** and **8C**) (23), our data suggest that the ability of LVS-infected cells to replenish their glycogen stores 12-24 hpi is critical to their longevity, and as such suggests that glycogen abundance may be a key determinant that dictates PMN lifespan.

**Figure 10:**
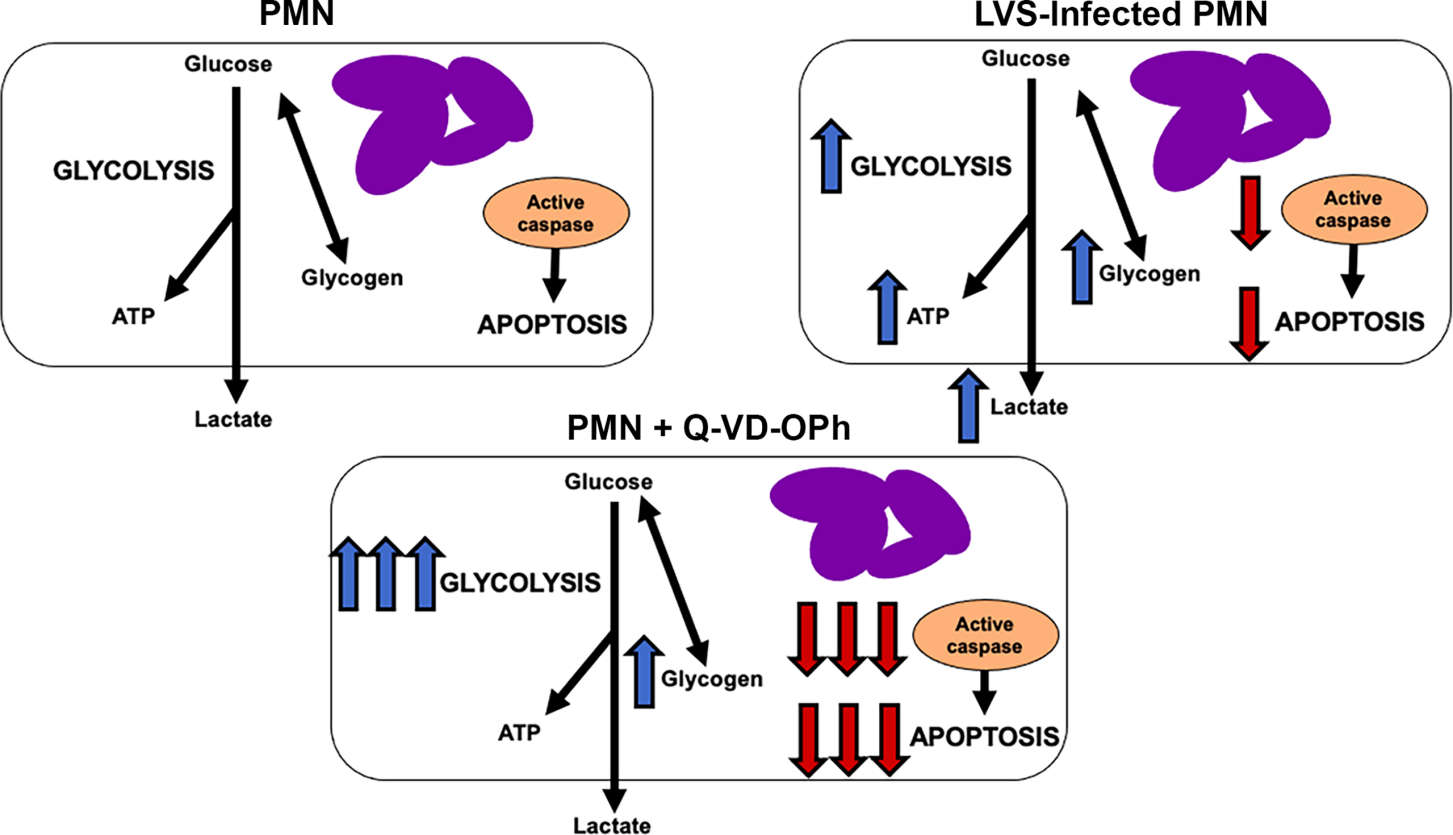
Model. Effects of *F. tularensis* and Q-VD-OPh on neutrophil metabolism. See Discussion for details.

The results of this study demonstrate that glucose metabolism and glycolysis are critical for PMN survival. We extend prior results of Healy et al. (47) to show that blockade of glycolysis with 2-DG and replacement of glucose with pyruvate significantly accelerated apoptosis of both control and LVS-infected cells. At the same time, the dynamic changes in glycogen stores reported here and the survival-enhancing effects of CP-91149 confirm that glycogen is critical for PMN viability (26), which contrasts markedly with the ability of CP-91149 to cause death of hepatocellular carcinomas (48). Finally, the fact that Q-VD-OPh-mediated caspase inhibition was sufficient to profoundly enhance glycolysis and glycolytic capacity and markedly increased glycogen stores in parallel with ablation of apoptosis at 24 hr (**Figure 9**) is additional definitive evidence that PMN metabolism and lifespan are intimately linked. These data are summarized in **Figure 10**.

Thus far, two direct links between glycolysis and apoptosis have been described. First, HK2 translocates to mitochondria and binds VDAC which enhances mitochondrial integrity, diminishes cytochrome *c* release and reduces sensitivity to BAX and other pro-apoptosis BCL-2 proteins (49, 50). Mitochondrial association of HK correlates directly with increased glycolytic flux and this may be regulated by phosphoinositide 3-kinase and Akt pro-survival signaling (49). Second, PKM2 has been linked to apoptosis resistance *via* its ability to phosphorylate and stabilize pro-survival BCL-2 family proteins (51, 52). PKM2 also translocates into the nucleus to modulate NF-κB-dependent gene expression and may inhibit caspase-3 indirectly *via* effects on miR-143 (51, 53). Our published data show that mitochondrial integrity is significantly prolonged by LVS infection, and in keeping with this, BAX mRNA and protein are diminished and BAX translocation to mitochondria is significantly delayed (22, 24), but whether this is attributable to HK2-VDAC interactions at mitochondrial surfaces and/or phosphoinositide 3-kinase signaling remains to be determined. In addition, several anti-apoptosis genes of PMNs are regulated by NF-κB including *BCL2A1, A20, XIAP* and *MCL1* (9). Caspase activation is inhibited in LVS infection by the concerted actions of XIAP, cIAPs and calpastatin (22, 24). *PKM2* expression is induced by LVS, but its specific role in our system is currently unknown. Conversely, *HK* expression is downregulated during apoptosis and caspases inhibit PFK and pyruvate kinase to block glycolysis during cell death (8, 54). To gain further insight into links between glycolysis and apoptosis we utilized western blotting to elucidate the effects of 2-DG on caspase-3 processing and the abundance of XIAP and MCL-1. Although procaspase-3 processing was enhanced, XIAP and MCL-1 were unchanged (**Supplementary Figure 8**). Thus, another possibility that must be considered is the ability of 2-DG to trigger cell death by inducting oxidative stress (55). Addressing this question in greater detail is of interest but is beyond the scope of this study.

Lactate is a major end product of glycolysis and is emerging as a regulator of metabolism with complex, cell type-specific effects (56–58). Similar to pyruvate, lactate can be used for gluconeogenesis, but its major established role is as a signaling intermediate that functions inside and between cells to alter function and activation state. For example, in monocytes, macrophages and T lymphocytes, elevated levels of lactate inhibit glycolysis and have been linked to immunosuppression as indicated by impaired production of proinflammatory cytokines by monocytes, changes in macrophage gene expression leading to M2 polarization, and impaired CD8+ T cell proliferation and toxicity (57, 58). In contrast, lactate signaling in tumor cells increases glucose uptake, enhances expression and activity of glycolysis enzymes and reduces mitochondrial function *via* a mechanisms linked to HIF-1α and *PIK3CA* (56). With regard to neutrophils, 10 mM exogenous lactate is sufficient to induce NETosis, but its effects on other aspects of cell function are unknown (56, 59). Our data indicate that the average amount of lactate released by LVS-infected PMNs over 24 hr (4.7 mM) had no discernable effect on the rate of constitutive apoptosis in the absence of infection (**Supplementary Figure 7**) and did not induce NETs (data not shown).

Although the apoptosis differentiation program of neutrophils has been extensively studied, the intracellular trigger that initiates changes in gene expression that lead to cell death is unknown. Recently, Sadiku et al. showed that the capacity to synthesize glycogen is required for neutrophil survival in mice and that blockade of glycogen breakdown with CP-91149 in glucose-free medium causes PMN death within 12 hr (26). The notion that glycogen levels directly control neutrophil longevity is also supported by our data which demonstrate that inhibition of glycogen breakdown by CP-91149 expanded glycogen stores and extend neutrophil lifespan (**Figures 8A–C**). At the same time, the data in **Figure 7** lead us to speculate that depletion of glycogen stores may trigger constitutive PMN death. Precisely what accounts for this remains to be determined, as GYS1, UGP2 and GBE1 enzymes and mRNA are unchanged (**Supplementary Figure 6**). A comparison of the data in **Figure 8** and **Supplementary Figure 3** confirms that although pyruvate can be used for gluconeogenesis it is less able to sustain glycogen stores, ATP production and PMN viability than glucose (26).

Glucose-6-phosphate that is not used for glycolysis or stored as glycogen can be transported into the ER. Mutations in *G6PT* prevent glucose-6-phosphate uptake into the ER, are associated with apoptosis and neutropenia in patients, and can be mimicked by treating wild-type cells with the G6PT inhibitor chlorogenic acid (60). However, Veiga-da-Cunha et al. demonstrated that PMN death is not due to impaired G6P transport, as has long been believed (61). Rather, toxicity is due to a long-lived common dietary glucose analog, 1,5-anhydroglucitol (1,5AG), that is abundant in blood and serum and is converted to 1,5AG6P by HK after uptake. In the absence of functional G6PT, 1,5AG6P cannot be further metabolized and accumulates, leading to inhibition of HK and PMN death. The lack of effect of chlorogenic acid on PMN viability in our hands (**Supplementary Figure 5**) supports this model as all our studies of *F. tularensis* and PMNs are performed using serum-free RPMI-1640 that is devoid of 1,5AG (22–24, 34).

The major metabolic pathways of neutrophils are aerobic and anaerobic glycolysis, TCA cycle, PPP, fatty acid biosynthesis and oxidation, glycogenolysis, glycogenesis, glutaminolysis, and in immature cells oxidative phosphorylation (7, 26, 28). These pathways are differentially utilized by PMNs under different conditions, and at least seven metabolic states have been described that distinguish 1) immature and tumor-associated neutrophils, 2) resting, mature PMNs, 3) cells undergoing chemotaxis, 4) phagocytosis, 5) canonical activation and ROS production, 6) elaboration of NETs or 7) apoptosis (8, 25, 28, 31, 46). Although additional studies are needed, the metabolic profile of LVS-infected PMNs described here is distinct from other metabolic profiles described to date. Specifically, the major metabolic pathways used by immature and tumor associated PMNs are fatty acid oxidation, TCA cycle and oxidative phosphorylation, whereas resting mature cells rely on glycolysis and phagocytosis selectively stimulates glycogenolysis. Chemotaxis is driven by glycolysis and mitochondrial purinergic signaling, NET formation requires glycolysis and PPP, and canonical activation is notable for glycolysis, glycogenolysis, PPP, TCA and glutaminolysis, whereas glycolysis and glycogenin synthesis are downregulated during apoptosis (8, 25, 28). In contrast, LVS-infected PMNs are notable for sequential glycogenolysis and glycogenesis, as well as induction of glycolysis and glucose uptake and downregulation of the PPP. As the PPP supplies NADPH that is essential for production of superoxide during the respiratory burst, downregulation of PPP enzymes and intermediates (**Figure 4**) (22) is in keeping with the ability of *F. tularensis* to elicit rapid, global inhibition of superoxide production *via* effects on NADPH oxidase assembly and activation (16, 17).

Metabolic reprogramming does not occur during or following every phagocytic event or infection, despite phagocytosis consuming half of a neutrophil’s total ATP (6). Kobayashi et al., demonstrated that although glycolysis genes are expressed in PMNs at rest, most are not differentially expressed during constitutive or phagocytosis-induced apoptosis, though expression of *HK* and genes linked to glycogen synthesis declined (8). Among other bacterial pathogens that persist or replicate in neutrophils, there are relatively little data regarding their effects on glycolysis or other aspects of host cell metabolism. Like *F. tularensis*, *Anaplasma phagocytophilum*, *Chlamydia pneumoniae* and *Neisseria gonorrhoeae* replicate intracellularly and modulate apoptosis (62–64). However, effects of *C. pneumoniae* and *N. gonorrhoeae* on expression of genes linked to glycolysis have not been reported, and although *A. phagocytophilum* elicits upregulation of *PFKFB3* and downregulation *LDHA*, consequences for cell metabolism, if any, remain to be determined (62). On the other hand, the periodontal pathogen *Filifactor alocis* modulates cholesterol homeostasis and selectively increases expression of genes linked to glycogen synthesis and glycosphingolipid metabolism rather than glycolysis (65). Upregulation of glycolysis has been observed in the context of infection with *Legionella pneumophila* or *Leishmania donovani* (27, 66) and LPS stimulation *in vitro* (26), but in these models glycolysis is coupled to PMN activation and bacterial clearance which contrasts sharply with the ability of *F. tularensis* to evade oxidative host defense, escape the phagosome and replicate in PMN cytosol (16, 17, 23).

The bacterial factors that mediate PMN metabolic reprogramming remain to be determined. Our published data demonstrate that maximum extension of neutrophil lifespan is independent of capsule and LPS and is mediated by the combined effects of intracellular bacteria along with *F. tularensis* lipoproteins and other factors that are rapidly released into conditioned media (22, 23, 34). Thus, it is tempting to speculate that bacterial lipoproteins and other secreted factors may begin to influence PMN metabolism, including *PFKL* expression, at the earliest stages of infection, as noted above. Addressing this knowledge gap is the focus of on-going studies by our group.

In summary, neutrophil turnover is disrupted during *F. tularensis* infection and, consequently, neutrophils contribute distinctly to tularemia pathogenesis by exacerbating host tissue destruction. Herein, we extended our previous studies of apoptosis inhibition to demonstrate neutrophil metabolic reprogramming by *F. tularensis*. We show that this bacterium elicits a distinct metabolic signature in human neutrophils that differs from other stimuli studied date. At the molecular level, this response is notable for induction of glycolysis, elevated lactate/pyruvate ratios, and complex glycogen dynamics. Although many questions remain unanswered, and other pathways and intermediates need to be explored, our findings reinforce links between metabolism and PMN longevity and set the stage for additional studies that may include other infections and disease states.

## Data Availability Statement

The raw data supporting the conclusions of this article will be made available by the authors, without undue reservation.

## Ethics Statement

The studies involving human participants were reviewed and approved by Institutional Review Board for Human Subjects at the University of Iowa. The patients/participants provided their written informed consent to participate in this study.

## Author Contributions

L-AA conceived of the study, designed experiments, analyzed data and co-wrote the manuscript. SK designed and performed experiments, analyzed data and co-wrote the manuscript. All authors contributed to the article and approved the submitted version.

## Funding

This work was supported, in part, by VA Merit Review grant 1I01BX002108, National Institutes of Health NIAID R21 AI137468-01 and R01 AI119965, all awarded to L-AA. SK is the recipient of a University of Iowa Post-Comprehensive Exam Fellowship.

## Conflict of Interest

The authors declare that the research presented here was conducted in the absence of commercial or financial relationships that could be construed as potential conflicts of interest.

## Publisher’s Note

All claims expressed in this article are solely those of the authors and do not necessarily represent those of their affiliated organizations, or those of the publisher, the editors and the reviewers. Any product that may be evaluated in this article, or claim that may be made by its manufacturer, is not guaranteed or endorsed by the publisher.
