# Supplementary material for: Metabolic Reprogramming Mediates Delayed Apoptosis of Human Neutrophils Infected With Francisella tularensis
Source: Front Immunol. 2022 May 25;13:836754. doi: 10.3389/fimmu.2022.836754 (PMC9174434; doi:10.3389/fimmu.2022.836754)
Supplement: Supplementary file 1 [file DataSheet_1.pdf]

SUPPLEMENTARY MATERIALS

Supplementary Table 1. Glycolytic and glycolytic enzyme gene primer-pair sequences.

Gene	Forward Sequence	Reverse Sequence
SLC2A3	TGCCTTTGGCACTCTCAACCAG	GCCATAGCTCTTCAGACCCAAG
HK2	GAGTTTGACCTGGATGTGGTTGC	CCTCCATGTAGCAGGCATTGCT
PFKFB3	GGCAGGAGAATGTGCTGGTCAT	CATAAGCGACAGGCGTCAGTTTC
PFKL	AAGAAGTAGGCTGGCACGACGT	GCGGATGTTCTCCACAATGGAC
LDHA	GGATCTCCAACATGGCAGCCTT	AGACGGCTTTCTCCCTCTTGCT
GBE1	GCCTTGACTTACCTCATGTTGGC	AGCACAGAGCTGGCATTCCTGA
UGP2	GCAGGAGCAAAATGCCATTGACA	CAGAAAACGGCTCCTTGGCACA
GYS1	CCGCTATGAGTTCTCCAACAAGG	AGAAGGCAACCACTGTCTGCTC

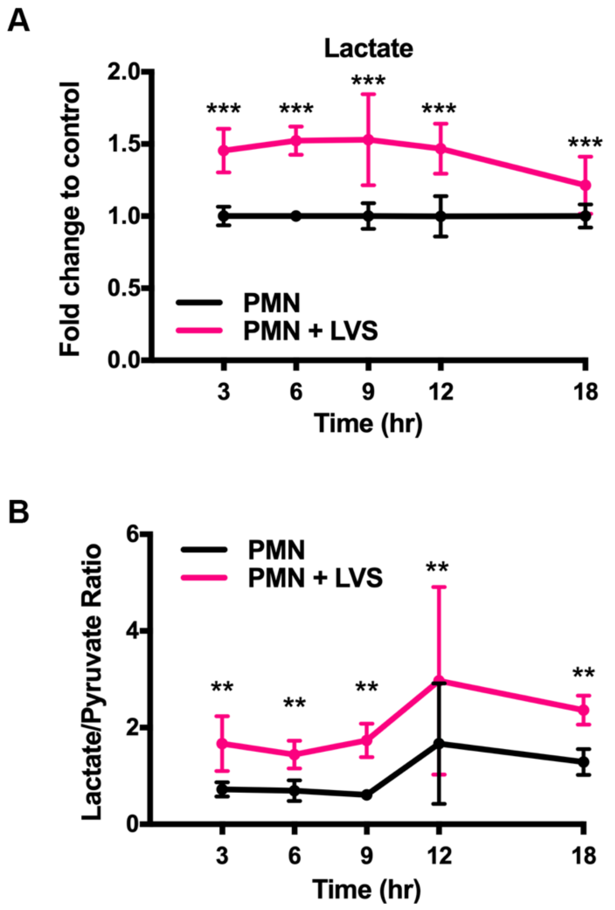

Supplementary Figure 1. LVS-infected PMNs contain significantly more intracellular lactate. (A-B) Lactate abundance (A) and lactate/pyruvate ratios (B) measured by GC-MS in control and LVS-infected PMNs at the indicated timepoints, n=3-6. ** $p < 0.01$, ***$p < 0.001$ compared to control PMNs at each timepoint. Where not visible, error bars are smaller than symbols.

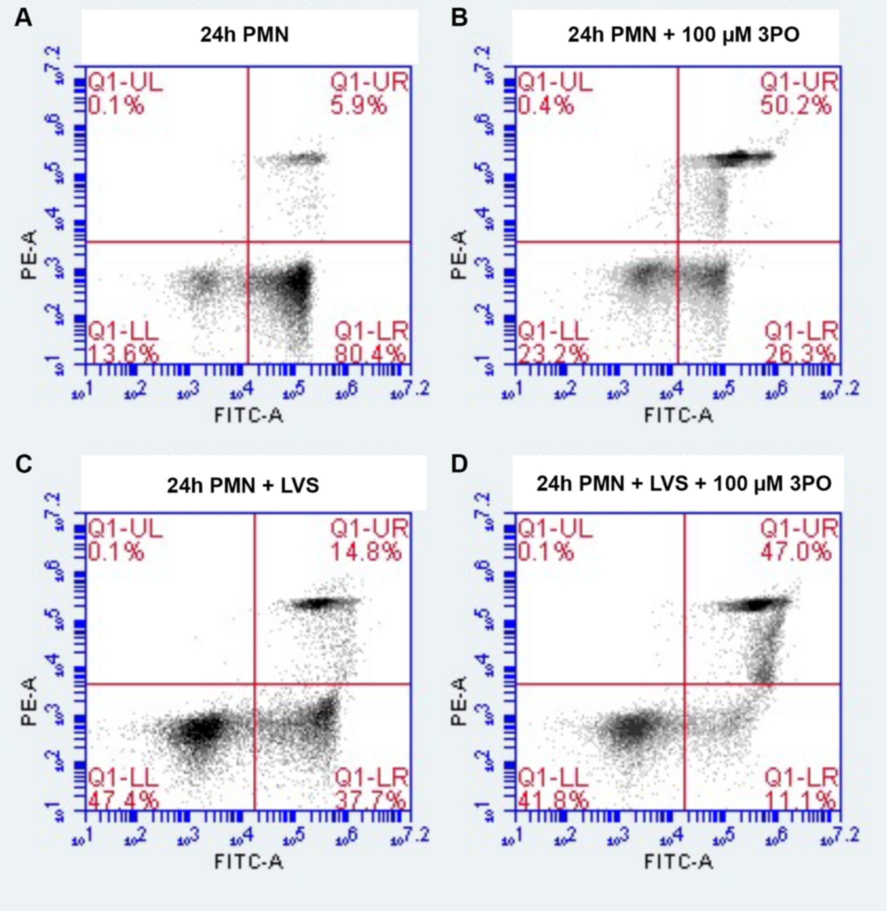

Supplementary Figure 2. Flow cytometry dot plots associated with Figure 5B. Flow cytometry dot plots of PMNs stained with Annexin V-FITC and PI at 24 hr. **(A)** PMNs. **(B)** PMNs treated with 100 μM 3PO. **(C)** LVS-infected PMNs. **(D)** LVS-infected PMNs treated with 100 μM 3PO. Data shown are representative of three independent determinations.

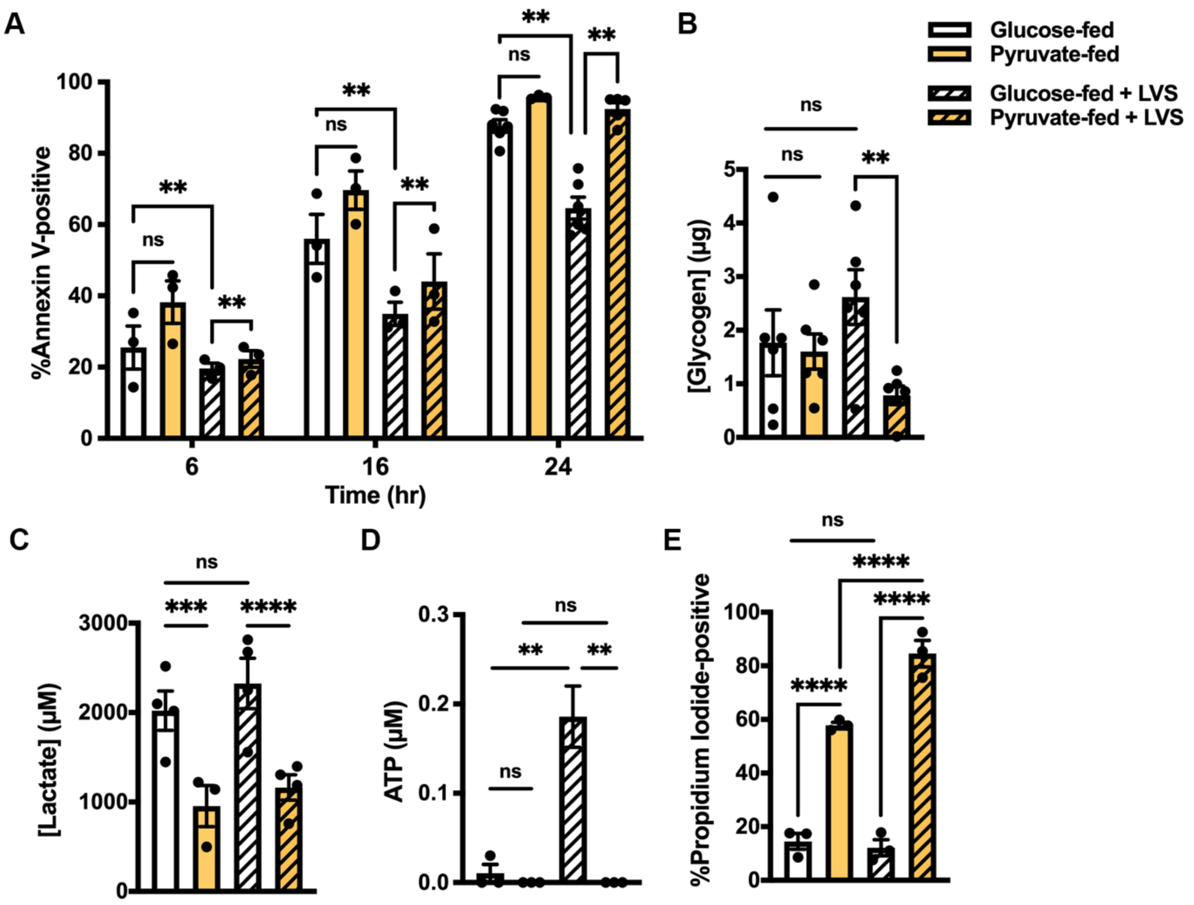

Supplementary Figure 3. Differential effects of forced pyruvate feeding on apoptosis, glycolysis, and glycogen abundance. (A) Measurement of apoptotic uninfected and LVS-infected PMNs in the presence of either 2 g/L glucose or 2 g/L pyruvate using Annexin V-FITC/PI staining and flow cytometry at the indicated timepoints, n=3-6. **$p < 0.01$, ns, not significant, as indicated. **(B)** Glycogen abundance in uninfected and LVS-infected PMNs in the presence of either 2 g/L glucose or 2 g/L pyruvate at 6 hr, n=6. **$p < 0.01$, ns, not significant, as indicated. **(C)** Amount of lactate released by control and LVS-infected PMNs in the presence of either 2 g/L glucose or 2 g/L pyruvate at 6 hr, n=3-4. ***$p < 0.001$, ****$p < 0.0001$, ns, not significant, as indicated. **(D)** ATP levels in uninfected and LVS-infected PMNs in the presence of either 2 g/L glucose or 2 g/L pyruvate at 24 hr, n=3. **$p < 0.01$, ns, not significant, as indicated. **(E)** Percentage of Annexin V-FITC positive cells that are also PI-positive at 24 hr, n=3-6, ****$p < 0.0001$.

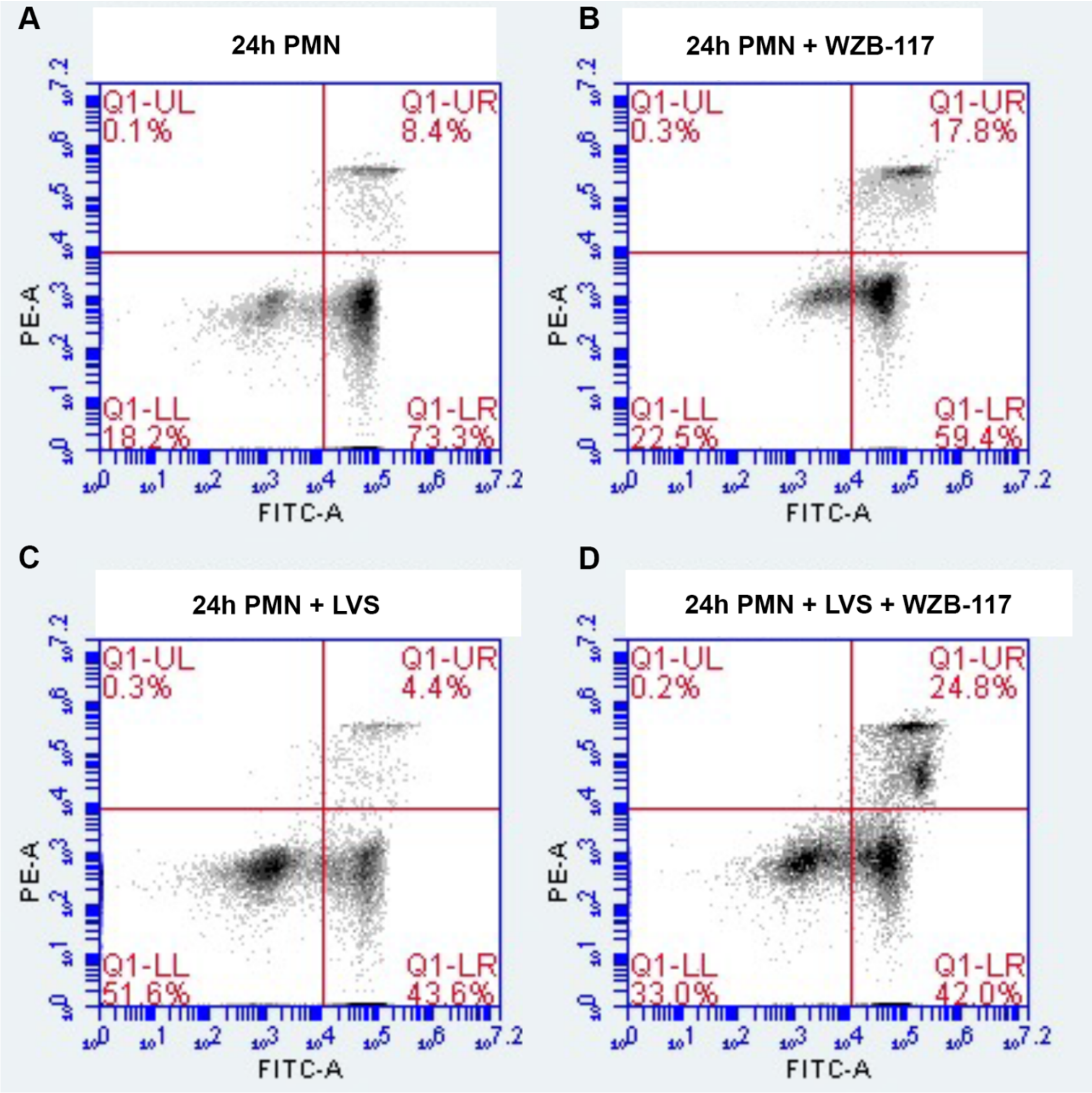

Supplementary Figure 4. Flow cytometry dot plots associated with Figure 6C. Flow cytometry dot plots of PMNs stained with Annexin V-FITC and PI at 24 hr. **(A)** PMN. **(B)** PMNs treated with 20 μM WZB-117. **(C)** LVS-infected PMNs. **(D)** LVS-infected PMNs treated with 20 μM WZB-117. Data shown are representative of four independent determinations.

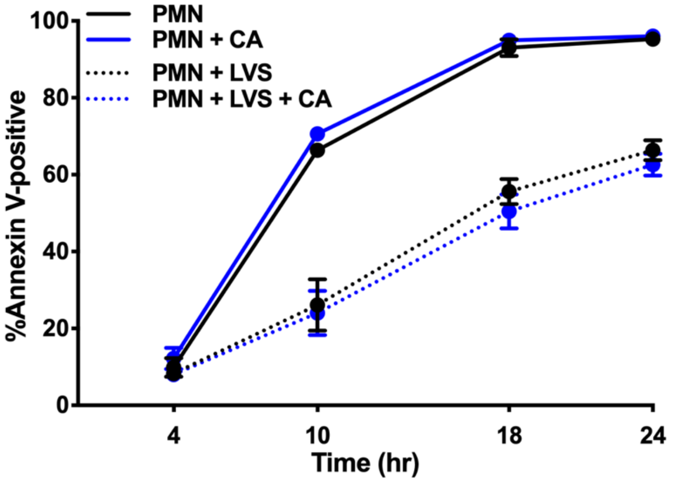

Supplementary Figure 5. G6PT inhibition does not impact PMN lifespan. Effect of chlorogenic acid (CA) on apoptosis of control and LVS-infected PMNs was measured using Annexin V-FITC staining and flow cytometry at the indicated time points, n=3-5.

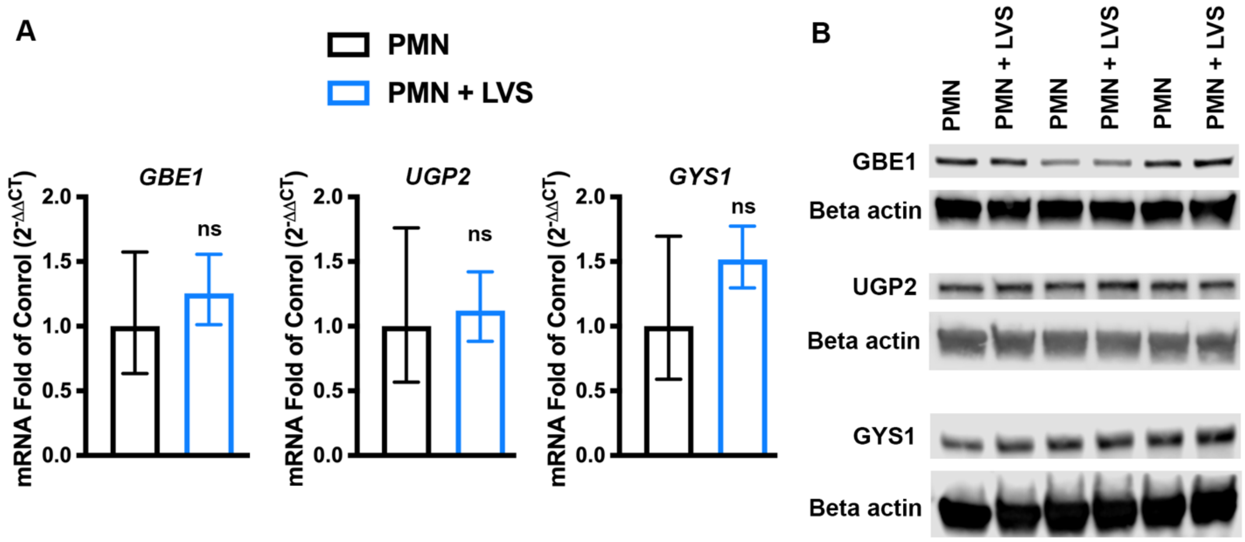

Supplementary Figure 6. LVS infection does not alter expression of glycogenesis enzyme genes or protein abundance. (A) qRT-PCR analysis of *GBE1*, *UGP2* and *GYS1* at 12 hr, n=3, ns, not significant. **(B)** Immunoblotting analysis of GBE1, UGP2 and GYS1 in neutrophil lysates from three different donors prepared at 12 hr, with β-actin as the loading control, n=3.

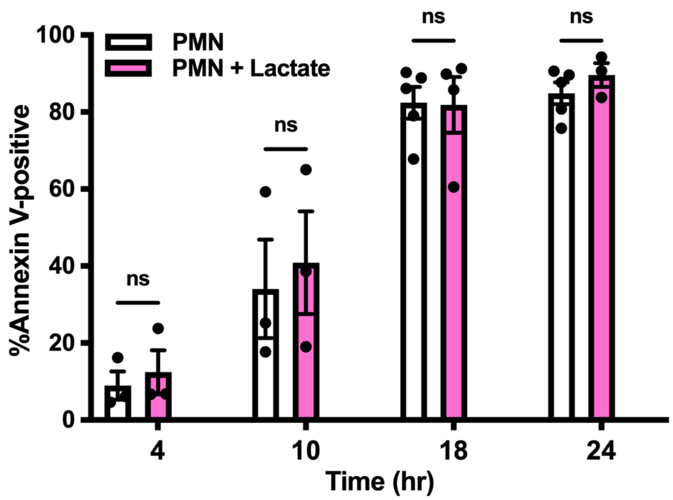

Supplementary Figure 7. Exogenous lactate does not alter PMN apoptosis kinetics. Neutrophils were incubated in medium with or without 4.7 mM sodium lactate supplementation, and the rate of apoptosis was quantified using Annexin V-FITC staining and flow cytometry. Data shown are the mean ± SEM, n=3. ns, not significant.

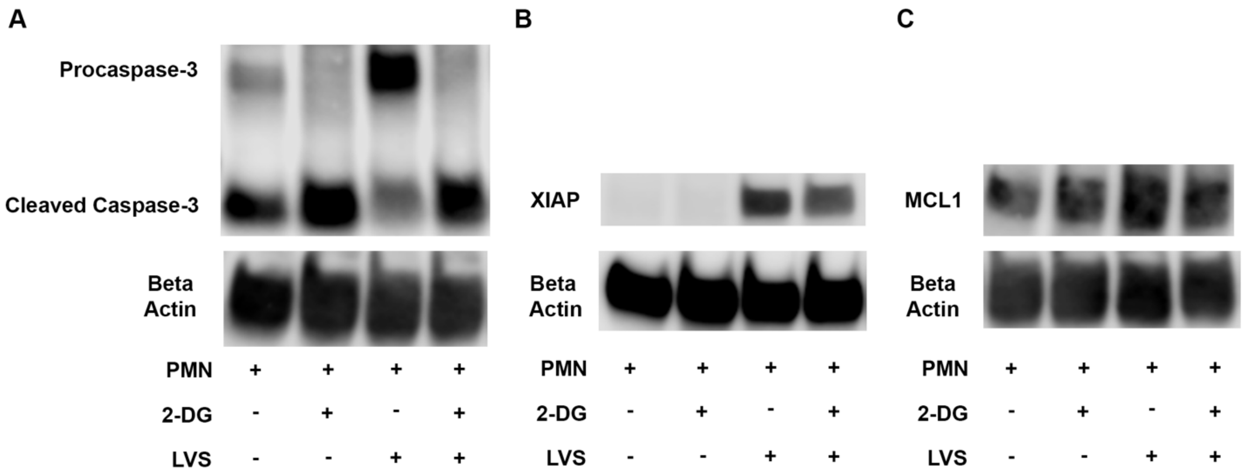

Supplementary Figure 8. Effects of 2-DG on caspase-3 processing and abundance of XIAP and MCL-1. Neutrophils were left untreated or infected with LVS in the presence or absence of 2-DG for 24 hr. Immunoblots of cell lysates were probed to detect caspase-3 (pro and mature forms) **(A),** XIAP **(B)**, and MCL-1 **(C)**. β-actin was used as the loading control. Data shown are representative of three independent determinations.
